# Objectively measured sedentary behavior and physical activity of Finnish 7- to 14-year-old children– associations with perceived health status: a cross-sectional study

**DOI:** 10.1186/s12889-016-3006-0

**Published:** 2016-04-16

**Authors:** Pauliina Husu, Henri Vähä-Ypyä, Tommi Vasankari

**Affiliations:** The UKK Institute for Health Promotion Research, Tampere, Finland

**Keywords:** Accelerometer, Pupils, Self-reported health, Sedentariness

## Abstract

**Background:**

Regular physical activity (PA) forms the basis for healthy growth and development. High volume of sedentary behavior (SB) on the other hand is harmful for health. The purpose of the study was to describe objectively measured PA and SB in Finnish school children. Furthermore, the study aimed at analyzing the association between PA, SB and perceived health status.

**Methods:**

The participants of this cross-sectional study were 7- to 14-year-old girls and boys (*n* = 1029), whose PA and SB during waking hours were measured with Hookie AM 20-accelerometer (Traxmeet Ltd, Espoo, Finland) for seven days. Perceived health status was assessed by a questionnaire. Association between PA, SB and health status was analyzed by logistic regression analysis using SPSS (Inc, Chicago IL).

**Results:**

Participants (age 10.3 ± 1.9, 52 % girls) with sufficient accelerometer data (at least 4 days with ≥ 10 h/day) were included into the study (*n* = 851, 88 %). The average measurement time was 13 h 27 min per day.

Participants spent on average 54 % (7 h 18 min) of waking hours sedentary, mainly sitting. They stood still on average 9 % of the time (1 h 15 min). Light PA covered on average 18 % (2 h 24 min) and moderate-to-vigorous PA 19 % (2 h 30 min). Younger participants and boys were more active than the older ones and girls.

Most (63 %) of the participants perceived their health status as excellent. In an adjusted logistic regression analysis greater sedentary time decreased the odds for excellent perceived health status (*p* = 0.001). In contrast higher number of steps per day (*p* = 0.019) increased the odds.

**Conclusions:**

Participants spent over half of their waking hours sedentary. Less SB and more steps were associated with excellent perceived health. There is a need for dose-response analyses between objectively measured PA and SB and specific health outcomes in children and adolescents. Also patterns of objectively measured PA and SB should be studied in more detail.

## Background

There is strong evidence showing that regular physical activity (PA) in childhood and adolescence is essential for healthy growth and development [1]. Recently, it has been suggested that a high volume of time spent in sedentary behavior (SB), especially sitting, may be harmful for health and wellbeing [2]. Based on objective measurement (accelerometry) of PA, recent large-scale studies have shown that adult populations spend most of their waking hours sedentary (55–62 %) and moderate-to-vigorous PA (MVPA) covers only a slight proportion of the day (4–6 %) [3–5]. Current evidence among children and adolescents show a corresponding trend. For example, most of the waking hours are spent sedentary and the amount of objectively measured PA decreases with increasing age [6, 7]. Only a minority of adolescents in Finland [7] and in other countries [8] meet current recommendations of PA having MVPA at least 60 min per day [9]. According to both objectively measured [6] and subjectively assessed [8] findings boys are on average more active than girls and younger children accumulate more MVPA than adolescents.

Perceived health status is an indicator of general health status [10]. Physical health is the strongest predictor of adolescents’ perceived health status, but besides that the perception includes also personal, socio-environmental, behavioral and psychological factors [11]. Young people tend to base their health perception mainly on health behaviors (e.g. diet, PA, alcohol and tobacco) [12]. Of these behaviors PA and SB have been shown to be independently associated with perceived health status, the association between PA and health status being positive and that between SB and health negative [13]. Herman et al. [14] analyzed the association between objectively assessed PA and SB and perceived health status and they reported that PA was positively associated with health status in boys, while SB was more important among girls.

Previous studies analyzing the association between PA, SB and perceived health have used mostly self-reported PA and SB levels [13]. Objectively analyzed associations have been conducted by count-based accelerometers [14], which may have limitations in identifying SB accurately [15]. Since objective measurement of PA and SB, mainly using accelerometers, has gained popularity and showed validity in youth populations [16, 17], there is an obvious need to confirm previously reported associations with newly validated methods. To our knowledge this is the first population-based study describing objectively measured SB and PA among school children using a tri-axial accelerometer using raw data. Thus, the primary purpose of the present study was to describe levels of objectively measured SB, standing still and PA using novel and recently validated analysis algorithms [16, 18] in Finnish school children. A secondary aim was to examine the association between SB, standing still and PA with perceived health status.

## Methods

The study is based on the Naantali Schools on the Move project, which is conducted in collaboration with the nationwide Finnish Schools on the Move programme [19]. The purpose of the project is to analyze the effects of the programme on PA, SB and fitness among school children of one study region targeting objective measurements of PA, SB and fitness for all school children in that region. The present study reports the results of the baseline data. In 2013 all school children from 12 schools attending the 1st to 7th grades (7–14 years of age) in Naantali and Masku municipalities in the South-West Finland were invited to participate in the project via their own school (*n* = 2060). All twelve schools were willing to participate the project, but only half of the invited pupils (*n* = 1029) agreed to participate the baseline measurements of the project during the spring and autumn terms in 2013. PA and SB data were collected with Hookie AM 20-accelerometer (Traxmeet Ltd, Espoo, Finland), which has been shown to be a valid measurement tool both among adults [20] and young people [16]. Teacher delivered the accelerometers during school-hours and gave both oral and written instructions for the use. One week later the devices were returned back to the teacher.

The accelerometer was attached to a flexible belt and participants were instructed to wear the belt around their waist for seven consecutive days during waking hours, except during shower and other water activities. The accelerometers collected and stored tri-axial data in raw mode in actual g-units. The data was analyzed in 6 s’ epoch length. PA was categorised into three intensity categories based on metabolic equivalents (MET): light, moderate and vigorous. Light PA was defined as activity corresponding 1.5 − 2.9 MET, moderate activity as 3.0–5.9 MET and vigorous activity 6 MET and over [18]. To be included into present study the participants had to have used accelerometers for at least four days, at least 10 h each day. Most of the children who met this criterion had used the device 6 to 7 days during a week including at least one weekend day. In the final results moderate and vigorous activities were combined (MVPA) since vigorous PA covered very slight proportion of the total recording time. Variables of SB and PA are presented either as proportions of total recording time (total time in each activity divided by the total recording time on proper measurement days) or as mean time in each activity during measurement days (total time in each activity divided by the number of measurement days). Compliance with PA recommendation was assessed in terms of the proportion of participants having at least 60 min of MVPA each day.

Steps were identified from the vertical impacts of the foot strikes with similar method described by Ying et al. [21]. For step detection was used a fixed threshold, which requires about 3 km/h walking speed to detect every step. The vertical component of the acceleration was calculated using an algorithm developed by Mizell [22]. According to definition of SB [23], time spent in sitting and reclining positions were combined to indicate SB, while standing still position was analysed separately. It is possible to accurately determine whether the participant is standing, sitting or lying by applying the information from the raw data of the three measurement axes of the accelerometer. While the body position during walking is upright and the direction of Earth’s gravity vector is constant, the orientation of the accelerometer for upright body position can be identified during normal walking. This known orientation can then be used as a reference value. Different body postures can be determined by calculating the angle between reference orientation and measured orientation. In standardized conditions, standing can be separated from sitting or lying with 100 % accuracy, and sitting from lying with 95 % accuracy [24]. Daily amount of stand-ups (breaks in sedentary time) was calculated on the basis of the number of lying/sitting periods ending-up with a clear vertical acceleration. Both steps and breaks in sedentary time are presented as mean values per measurement day (total number of steps and breaks divided by the number of measurement days).

Age, gender, perceived health status, health-related symptoms and participation in sport club activities were assessed by a questionnaire. The youngest children (1st to 3rd graders) filled the questionnaire together with their parents at home and the older ones (4th to 7th graders) filled the questionnaire at school with their teacher.

Perceived health status was assessed by asking children to report whether they perceived their health status as excellent, good, fair or poor. The corresponding question on health has been used in several other studies [10, 12]. Perceived symptoms were assessed by asking the participants to report whether they had neck and shoulder pain, low back pain, stomach pain, headache or tiredness during the past 6 months. The response alternatives were never or seldom, approximately once a month, approximately once a week and nearly every day. Sport club participation was assessed by asking the participants to report whether they had participated in PA organized by a sport club during the past six months. The response alternatives were no, yes sometimes and yes regularly.

Participants’ height and weight were measured by the same testing team with the Inbody 720- equipment (bioelectrical impedance analysis, Biospace Co., Ltd, Seoul, Korea). The body mass index (BMI) was calculated by dividing weight in kilograms by a square root of height in meters.

All data from the questionnaire, the calculated variables from bioelectrical impedance analysis and accelerometer analysis were combined using participant’s name and study number. The data was analyzed by SPSS software, version 22 (SPSS Inc, Chicago IL). The descriptive analyses were conducted separately for boys and girls according to school grade. Univariate analysis of variance (ANOVA) was used for height, weight and BMI. Perceived health, health-related symptoms, compliance with PA recommendation and sport club participation were analyzed by cross-tabulations with Somers’d test to indicate differences between all school grades and Chi^2^ test to indicate gender difference and difference between one school grade group and the other two groups. Effect sizes were expressed in terms of Cramer’s V for the categorical variables. Regarding perceived health differences were analyzed between excellent and good/fair/poor. Regarding health-related symptoms comparison was conducted between once a week/daily and once a month/seldom and sport club participation was categorized into never/sometimes and regularly.

The association between PA, SB and perceived health status was analyzed first by age- and gender-adjusted analysis of covariance (ANCOVA) and further by logistic regression analysis adjusted for gender, age, BMI and perceived health-related symptoms. For Table 3 MVPA was modified to indicate activity per 10 min (daily minutes divided by 10) in order to help interpretation of the results. At first the PA and SB variables were analyzed separately. The final models were conducted by selecting the most powerful indicators (*p* < 0.05) of perceived health status, and including these into a multivariable logistic regression analysis using backwards elimination method with likelihood ratio criteria and adjusted for gender and age (model 1), additionally for BMI (model 2) and further for perceived health-related symptoms (model 3).

### Ethics

Children and their parents or guardians were informed about the study beforehand. Participation in the study was voluntary, and participants had the right to drop out at any time without a specific reason. A written informed consent was obtained from the children and their parent or guardian before participation. Only children with a fully completed consent form were included in the study. The study has been performed in accordance with the Declaration of Helsinki and it has been approved by the Ethics Committee of the Tampere region (2/2013).

## Results

A total of ninety-four percent (*n* = 964) of the 1029 children who participated to the study were willing to wear accelerometer. Eighty-eight percent (*n* = 851) of those who wore the accelerometer wore it at least for four days, at least 10 h each day, which was used as an inclusion criteria for the present study. Those who did not meet the criteria were more likely to be boys (63.7 % vs. 48.3 %, *p* = 0.002) and less likely to have excellent perceived health status (51.6 % vs. 62.6 %, *p* = 0.042) than those who met the criteria. However, those not meeting the criteria did not differ from those meeting the criteria in terms of school grade (*p* = 0.277) or self-reported PA (*p* = 0.607).

Most (72 %) of the participants meeting the criteria had used accelerometer for 6 or 7 days during a week. The wearing time was on average 13 h 27 min per day. There was no gender difference in the wearing time. However older participants wore the accelerometer on average for longer time per day when compared to the younger ones.

Table 1 presents the general characteristics of the participants. Just over half (52 %) of the participants were girls. Nearly two-thirds of the participants perceived their health status as excellent. Older participants had on average poorer perceived health status than the younger ones. Tiredness and headache were the most typically reported health-related symptoms. Fourteen percent of the participants reported tiredness at least once a week. The corresponding percentage for headache was 12 %. The prevalence of neck and shoulder pain, headache and tiredness was lower among the 1st–3rd graders than among the older participants. Girls reported on average more neck and shoulder pain and stomach pain than boys. Most of the participants (69 %) reported that they participated in sport club activities regularly. Participation was more typical for younger participants compared to older ones. However, only one third of the participants met the recommendation for PA having MVPA for at least 60 min per day. Older participants were less likely to meet the PA recommendation than the younger ones.

**Table 1:** General characteristics of the participants. For the analyses perceived health was categorized into excellent vs. good/fair/poor, health-related symptoms were categorized into once a week/daily vs. once a month/seldom/never and sport club participation was categorized into never/sometimes vs. regularly

	School grade	1–3		4–6		7		p-value*		
	Gender	Boys	Girls	Boys	Girls	Boys	Girls	Boys	Girls	p-value**
	n	*n* = 206	*n* = 191	*n* = 174	*n* = 212	*n* = 31	*n* = 37	*n* = 411	*n* = 440	
	Height, cm (SD)	138.6 (9.6)	136.3 (7.3)	151.5 (8.2)	154.2 (8.5)	164.0 (9.1)	160.0 (6.1)	<0.001	<0.001	0.240
	missing (n)	24	21	15	26	7	5			
	Weight, kg (SD)	33.4 (7.1)	32.5 (7.2)	42.8 (10.3)	45.0 (10.1)	56.5 (15.3)	52.9 (9.1)	<0.001	<0.001	0.168
	missing (n)	24	21	15	26	7	5			
	BMI, kg/m^2^ (SD)	17.3 (2.4)	17.3 (2.7)	18.5 (3.2)	18.8 (3.1)	20.7 (4.0)	20.5 (3.0)	<0.001	<0.001	0.227
	missing (n)	24	21	15	26	7	5			
Perceived health, %	excellent	72.4	73.9	59.1	54.9	50.0	19.4	0.002	<0.001	0.160
	good	27.1	26.1	38.4	40.8	42.9	74.2			
	fair	0.5	0	2.4	2.9	7.1	6.5			
	poor	0	0	0	1.5	0	0			
	missing (n)	14	15	10	6	3	6			
	p-value***	0.003	<0.001	0.034	0.024	0.082	<0.001			
	effect size^a^	0.15	0.24	0.11	0.11	0.09	0.24			
Neck and shoulder pain, %	seldom/never	87.4	86.9	69.5	60.5	60.7	58.1	0.021	<0.001	0.024
	once a month	10.5	11.4	23.2	25.4	32.1	29.0			
	once a week	2.1	1.1	4.3	12.2	3.6	12.9			
	nearly daily	0	0.6	3.0	2.0	3.6	0			
	missing (n)	15	15	10	7	3	6			
	p-value***	0.016	<0.001	0.036	<0.001	0.536	0.393			
	effect size^a^	0.12	0.22	0.11	0.19	0.03	0.04			
Low back pain, %	seldom/never	93.7	94.9	83.5	79.5	82.1	67.7	0.102	0.226	0.261
	once a month	5.2	2.8	14.6	16.6	7.1	25.8			
	once a week	1.0	2.3	1.2	3.4	3.6	3.2			
	nearly daily	0	0	0.6	0.5	7.1	3.2			
	missing (n)	15	15	10	7	3	6			
	p-value***	0.155	0.276	0.759	0.574	0.001	0.329			
	effect size^a^	0.07	0.05	0.002	0.003	0.17	0.05			
Stomach pain, %	seldom/never	69.1	63.6	71.0	60.2	71.4	35.5	0.352	0.920	0.016
	once a month	26.7	26.7	24.1	30.6	17.9	54.8			
	once a week	4.2	8.0	3.7	8.3	7.1	9.7			
	nearly daily	0	1.7	1.2	1.0	3.6	0			
	missing (n)	15	15	12	6	3	6			
	p-value***	0.473	0.897	0.970	0.879	0.148	0.963			
	effect size^a^	0.04	0.01	0.00	0.01	0.07	0.00			
Headache, %	seldom/never	66.1	66.9	50.9	48.1	35.7	32.3	0.003	0.004	0.319
	once a month	25.5	23.4	33.1	32.5	35.7	45.2			
	once a week	7.8	8.6	14.1	15.0	28.6	16.1			
	nearly daily	0.5	1.1	1.8	4.4	0	6.5			
	missing (n)	14	16	11	6	3	6			
	p-value***	0.006	0.005	0.148	0.030	0.011	0.260			
	effect size^a^	0.14	0.14	0.07	0.11	0.13	0.06			
Tiredness, %	seldom/never	67.0	76.1	61.5	60.7	50.0	51.6	0.173	0.028	0.643
	once a month	23.0	15.3	29.8	25.7	17.9	25.8			
	once a week	9.4	5.7	8.7	11.2	28.6	19.4			
	nearly daily	0.5	2.8	0	2.4	3.6	3.2			
	missing (n)	15	15	13	6	3	6			
	p-value***	0.490	0.05	0.209	0.356	<0.001	0.063			
	effect size^a^	0.04	0.10	0.06	0.05	0.19	0.09			
PA recommendation met, %	yes	37.4	39.8	37.9	25.5	25.8	10.8	0.559	<0.001	0.052
(≥60 min MVPA per day)	p-value***	0.788	<0.001	0.668	0.029	0.189	0.007			
	effect size^a^	0.01	0.18	0.02	0.10	0.07	0.13			
Sport club participation, %	never	17.5	23.6	25.5	24.0	35.7	33.3	0001	0.073	0.573
	sometimes	4.6	4.5	10.9	7.8	7.1	16.7			
	regularly	77.8	71.9	63.6	68.1	57.1	50.0			
	missing (n)	12	13	9	8	3	7			
	p-value***	0.001	0.187	0.014	0.894	0.114	0.024			
	effect size^a^	0.17	0.07	0.13	0.01	0.08	0.11			

*MVPA* moderate-to-vigorous physical activity

*BMI* body mass index

*p-value for school grade differences: Analysis of variance for height, weight and BMI, Somers’d test for perceived health, health-related symptoms, physical activity recommendation and sport club participation

**p-value for gender difference: Analysis of variance for height, weight and BMI, Chi^2^test for perceived health, health-related symptoms, physical activity recommendation and sport club participation

***p-value for school-grade difference, one group vs. the other two groups: Chi^2^test

^a^Cramer’s V indicating effect size

**Fig. 1:**
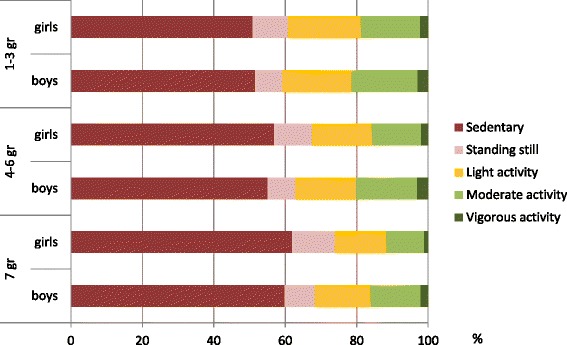
Sedentary behavior and physical activity as a proportion of measurement time

Participants spent over half (54 %) of their waking hours (7 h 18 min) sedentary, mainly sitting. Standing still covered on average 9 % of the time (1 h 15 min), light PA 18 % (2 h 24 min), moderate PA 16 % (2 h 11 min) and vigorous PA 3 % (20 min) (Fig. 1). The 1st–3rd graders spent on average higher proportion of measurement time in light (*p* < 0.001) and moderate (*p* < 0.001) PA than the 4th–6th graders and the 7th graders, and the 4th–6th graders had on average higher proportions than the7^th^ graders (*p* < 0.001). For vigorous PA, 7th graders had smaller proportions of activity than the 1st–3rd graders (*p* < 0.001) and the 4th–6th graders (*p* < 0.001), but these groups of younger children did not differ from each other (*p* = 0.406). The boys spent on average higher proportion of time in moderate and vigorous PA than the girls (*p* < 0.001), but there was no gender difference in the proportion of light PA (*p* = 0.149). Regarding the proportion of SB, girls were slightly more sedentary than the boys (*p* = 0.027) and the participants on higher school grades had on average higher proportions than those on lower grades (*p* < 0.001).

**Fig. 2:**
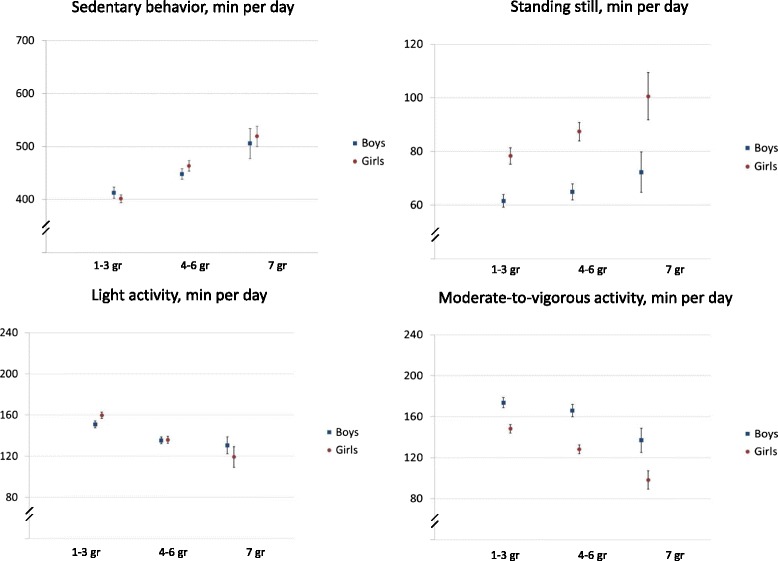
Title: Mean daily minutes of sedentary behavior and physical activity

Figure 2 presents the mean time in minutes spent in SB, standing still and PA separately for the boys and girls. Since vigorous PA was so scarce, it was combined to moderate PA indicating MVPA. School-grade groups differed statistically significantly from each other. The 7th graders were on average more sedentary (*p* < 0.001), stood more (*p* = 0.003) and had less light PA (*p* = 0.001) and less MVPA (*p* < 0.001) than the 4th–6th graders. Correspondingly, the 4th–6th graders were on average more sedentary (*p* < 0.001), stood more (*p* < 0.001) and had less PA (*p* < 0.001) than the 1st–3rd graders. There was no gender difference in SB (*p* = 0.209). However, girls stood on average more than the boys (*p* < 0.001). Boys accumulated more MVPA than girls in all grade-groups (*p* < 0.001), but for light PA there was no gender difference (*p* = 0.259). Boys took on average more steps per day than girls (14290 vs. 11658, *p* < 0.001), but girls had on average more breaks in SB than boys (47.7 vs. 43.4, *p* < 0.001) (data not shown). The 7th graders took on average fewer steps per day and had fewer breaks in SB than the 4th–6th graders (*p* < 0.001) and the 1st–3rd graders (*p* < 0.001).

Participants perceiving their health status as excellent took on average more steps per day and had on average more breaks in SB, fewer sedentary time and more MVPA than those who had good, fair or poor health status when gender and school grade were adjusted for (Table 2). Perceived health status was not associated with either standing time or light PA.

**Table 2:** Physical activity and sedentary behavior according to perceived health status. Legend: Gender- and school grade-adjusted means of daily values and 95 % Confidence intervals

	Perceived health status				
	Excellent		Good/Fair/Poor		
	mean	95 % CI	mean	95 % CI	p-value
Breaks in SB (number/day)	46.6	45.7-47.5	44.3	43.1-45.5	0.003
Steps (number/day)	13272	12996-13549	12448	12086-12810	0.001
SB (min/day)	429.5	423.7-435.4	450.1	442.5-457.7	<0.001
Standing still (min/day)	75.6	73.7-77.6	72.6	70.1-75.1	0.068
Light PA (min/day)	145.5	143.5-147.5	142.4	139.8-145.1	0.074
MVPA (min/day)	153.7	150.7-156.7	146.5	142.7-150.4	0.005

*CI* Confidence interval

*MVPA* moderate-to-vigorous physical activity

*PA* physical activity

*SB* sedentary behavior

When SB and PA variables were analyzed separately as potential predictors of excellent health status, gender and school grade adjusted analysis showed that all of them, except standing still, were statistically significantly associated with excellent perceived health (Table 3). When these single predictors were included into the same model, only sedentary time per day remained statistically significantly associated and number of steps per day remained nearly significant. Further adjustment for BMI decreased the strength of breaks in SB as a predictor of excellent health status. When all single predictors were included into the same model, higher sedentary time decreased the odds for excellent health status. In contrast, each 1000 steps per day increased the odds. Adjusting the models further by perceived health-related symptoms decreased the strength of MVPA as a significant predictor of excellent health, but the adjustment did not change the results of the final multivariable model.

**Table 3:** Associations of sedentary behavior and physical activity (mean of daily values) with perceived health status. Legend: Logistic regression analysis adjusted for gender and school grade (Model 1),for gender, school grade and body mass index (Model 2) and for gender, school grade, body mass index and health-related symptoms (Model 3) predicting excellent perceived health status

A	Model 1			Model 2			Model 3		
	OR	95 % CI	p-value	OR	95 % CI	p-value	OR	95 % CI	p-value
SB (h/day)	0.71	0.62-0.82	<0.001	0.76	0.66-0.88	<0.001	0.77	0.66-0.89	<0.001
Standing still (h/day)	1.42	0.94-2.16	0.100	1.46	0.93-2.32	0.103	1.48	0.93-2.37	0.099
Light PA (h/day)	1.88	1.27-2.77	0.002	1.70	1.11-2.61	0.016	1.69	1.08-2.65	0.021
MVPA (min per day/10)	1.08	1.03-1.13	0.001	1.05	1.00-1.11	0.043	1.05	1.00-1.11	0.061
Breaks in SB (number/day)	1.03	1.01-1.04	<0.001	1.01	1.00-1.03	0.086	1.01	0.99-1.03	0.190
Steps per day/1000 steps	1.10	1.05-1.16	<0.001	1.09	1.03-1.15	0.003	1.08	1.02-1.15	0.005
B	Model 1			Model 2			Model 3		
	OR	95 % CI	p-value	OR	95 % CI	p-value	OR	95 % CI	p-value
SB (h/day)	0.78	0.67-0.91	0.002	0.79	0.67-0.93	0.005	0.78	0.67-0.91	0.002
Steps per day/1000 steps	1.05	1.00-1.11	0.051	1.14	1.03-1.27	0.013	1.06	1.00-1.12	0.043
Breaks in SB (number/day)	1.02	1.00-1.03	0.061						
MVPA (min per day/10)				0.92	0.84-1.02	0.100			

*CI* Confidence interval

*MVPA* moderate-to-vigorous physical activity

*OR* odds ratio

*PA* physical activity

*SB* sedentary behavior

A = indicators of sedentary behavior and physical activity analyzed separately

B = statistically significant (*p* < 0.05) predictors included into the same model using backwards elimination method with likelihood ratio criteria

## Discussion

To our knowledge, this is the first study to describe objectively measured PA and SB in a large sample of Finnish school children using raw accelerometer data. The main results of the study were that participants spent over half of their waking hours sedentary, objectively measured SB decreased the odds for excellent perceived health status and higher number of steps per day was associated with excellent health status.

According to objective measurements of the present study PA covered only slightly over one third of the measurement time. Children spent over seven hours per day in a sitting or reclined position without movement. This is in accordance with previous studies using objective measurements. Matthews et al. [25] reported that American young people were sedentary 6–8 h per day. Furthermore, a corresponding amount of time spent in SB among a sample of Canadian children was 8.6 h (62 % of the waking hours) [6]. Consistent with the current study, adolescents in the Canadian study accumulated more sedentary time and less active time than the younger children [6]. A study conducted with English children aged 8–10 years old, suggested that they were more sedentary, SB covering on average 81 % of their waking hours [26]. UK is one of the first countries having recommendations for SB and encouragement to break up prolonged periods of sitting embedded in the national PA guidelines [27]. Also in Finland recommendation for PA of school-aged children includes a guideline to limit excessive sitting and screen time [28]. Although previous studies have reported that girls are more sedentary than boys [14, 26, 29, 30], present study did not find statistically significant gender difference in sedentary time.

In the present study both light PA and MVPA covered nearly one fifth of the recording time corresponding on average to 2.5 h. Thus, the total amount of activity in the present study was very similar to that of the Canadian study [6], although light PA was more (4 h/day) and MVPA less frequent (54 min/day) in the study by Colley et al. [6]. In that study, the Canadian children also took on average fewer steps than the participants of the present study (boys: 12121 vs. 14290 and girls 10327 vs. 11658). In both studies MVPA consisted mainly of moderate PA, vigorous activity covering only a slight proportion of recording time [6]. Boys of the present study accumulated on average more MVPA than girls, which is in line with previous findings [6, 29]. Younger children of the study were on average more active and less sedentary than the older ones. De Decker et al. [31] suggested that children’s PA peak sometime between 6 and 7 years of age and then starts to decline.

To accumulate recommended 60 min of MVPA [9] school children need to take on average 13000–15000 steps per day in boys and 11000–12000 steps per day in girls [32]. For adolescents (both boys and girls), the corresponding step amount is 10000–11700 [32]. Mean step counts of the present study (14290 in boys and 11658 in girls) fall well between these limits. Participants of the study accumulated on average 2.5 h of MVPA per day, but this activity was unequally distributed across measurement days. To meet the PA guideline participants need to have at least 60 min of MVPA every day [9] and based on this criterion, on average one third of the participants of the present study met the guideline. de Vries et al. [33] studied the fulfillment of this guideline among 6–11 year old children and reported that the proportion of children meeting the guideline varied according to guideline operationalization in terms of intensity, bout duration and days. Independent of guideline operationalization and assessment method (self-report, accelerometer) boys met the guideline better than girls, which is in line with the present findings. Also Konstabel et al. [30] reported corresponding gender difference; the proportion of boys meeting the guideline of at least 60 min of MVPA per day ranged from 10 to 34 % between eight European countries. In girls the corresponding range was 2–15 %.

Most of the participants (63 %) of the present study perceived their health status as excellent, which is in line with previous studies (52–71 %) [13, 14]. In our sample the proportion of children perceiving excellent health was lower among the older participants when compared to younger ones. Previous studies have reported decreased perceived health with increasing age [10, 11]. Additionally, it has been reported that girls rated their health as poorer than boys [10, 11], which was seen also in the present study. Poorer perceived health status among girls and older participants may reflect health-related symptoms reported (Table 1). The symptoms tended to increase from younger to older children. Furthermore, in Finland pupils typically change school after the 6th grade. The 6th grades are the oldest pupils in the elementary school while the 7th graders are the youngest ones in the secondary school. Such changes may affect the health perceptions of the 7th graders.

In line with Herman et al. [13] we found a positive association between PA and perceived health status and negative association between SB and health. Sedentary time and number of steps per day were the most powerful predictors of excellent health status in the adjusted analysis of the present study. This may suggest that general physical activity, despite of the intensity, is important for perceived health. Instead of MVPA minutes we conducted the analysis also by using the variable indicating compliance with MVPA recommendation as a potential predictor of health status, but there was no association (data not shown). Based on self-reported information Herman et al. [13] reported that PA and SB were independent predictors of perceived health. According to Herman et al. [14] physical self-esteem and body-image may be mediating factors in the associations between PA, perceived health and BMI. Previously it has been reported that higher BMI is associated with more sedentary time [26] and that obese children are more likely to report poorer than very good health than normal weight children [14]. This was true also in the present study sample (data not shown). Obesity has been linked with poor perceived health regardless of socioeconomic status or school type [34]. In the present study the potential role of BMI in the association between PA, SB and perceived health was controlled by adjusting for this outcome. While, this adjustment decreased the strength of breaks in SB as a predictor of excellent health status, overall it did not change the results of the study. Moreover, adjustment of the analysis further by perceived health-related symptoms did not change the main results.

The strengths of the present study were a large sample of school children and objective measurements of PA and SB using novel, validated analysis algorithms that were able to identify SB, standing still and different PA levels. The measured acceleration data was collected and saved as raw signals. Saved data was post processed using mean amplitude deviation algorithm. The analyzing method is device-independent allowing direct comparison between different studies [16, 18]. Participants wore accelerometers on average 13.5 h per day, which is very close to the mean wearing time reported by Colley et al. [6] (13.6 h). Girls and boys did not differ in wearing time, but older participants had on average longer wearing times than younger ones, which is also in line with previous findings [6]. The average recording time can be considered as representative of school children’s waking hours. Most children who had used accelerometers for at least four days and at least 10 h each day had used the device 6 to7 days during a week including at least one weekend day, which increases the representativeness of the data.

A weakness of this study is that the sample was drawn from only two municipalities in South-West Finland. Thus, the results may not be generalizable to the whole country. All school children from these municipalities attending 1st to 7th grades were invited to the study, but only half of them were willing to participate the study. The data was collected during spring and autumn and no data from mid-winter season was included. During winter the amount of PA is probably somewhat smaller than during the spring and autumn. These factors may further limit the representativeness of the findings. Furthermore, due to the cross-sectional nature of the study we cannot draw any causative conclusions of the results. Additionally, all participants were not willing to participate in BMI measurements or to rate their perceived health status, which caused some missing data.

In the future objectively measured PA and SB of school children should be studied in more representative study samples using prospective designs. Also dose-response relationship between objectively measured PA, SB and specific health outcomes warrant further studies. Furthermore, patterns of SB and PA should be studied in more detail.

## Conclusions

School children spent over half of their waking hours sedentary. Higher SB decreased the odds for excellent perceived health status. MVPA covered only a slight proportion of waking hours. Higher number of daily steps increased the odds of excellent health status. Thus, to promote health status of school children attention should be paid on decreasing their SB and increasing their daily activity. This could be achieved by integrating general PA and more steps into schooldays. Physical education classes form only a part of PA at school. Recesses between lessons and commuting may offer a great potential to increase PA and decrease SB among school children.

## Availability of data and materials

The data behind the results can be obtained for scientific use from the authors.

## Abbreviations

ANCOVA: analysis of covariance
ANOVA: analysis of variance
BMI: body mass index
MET: metabolic equivalent
MVPA: moderate-to-vigorous physical activity
PA: physical activity
SB: sedentary behavior
